# Policy Series: The Older Americans Act, the Aging Network, and the Pandemic

**DOI:** 10.1093/geroni/igab046.1163

**Published:** 2021-12-17

**Authors:** Brian Lindberg

## Abstract

This session provides insights into how the pandemic challenged the capabilities and ingenuity of the Older Americans Act (OAA) programs and the aging network and what it means for in-home and community aging services now and in the future. Speakers will include key aging network stakeholders, who will discuss the overnight evolution of programs serving often isolated older adults.

